# Supplementary material for: Reproductive system, temperature, and genetic background effects in experimentally evolving populations of Caenorhabditis elegans
Source: PLoS One. 2024 Apr 1;19(4):e0300276. doi: 10.1371/journal.pone.0300276 (PMC10984399; doi:10.1371/journal.pone.0300276)
Supplement: S1 Appendix — (DOCX) [file pone.0300276.s001.docx]

**S1 Appendix**

The variance of an individual proportion in fitness assays is:

$$var\left( p_{ij} \right)=\frac{p_{i}(1-p_{i})}{N_{j}}$$

where *p_ij_* is the proportion of focal individuals in offspring (*i* = 1) and parental (*i* = 0) generation of *j*-th fitness assay, *N_j_* is the total number of individuals in a given assay batch.

Then, across all fitness assay repeats, the overall variance of aggregated proportions can be derived as:

$$var\left( \hat{p_{ij}} \right)=\frac{\sum_{j} {var(p}_{ij})+2\sum_{j} \sum_{k>j} cov\left( p_{ij}p_{ik} \right)}{n^{2}}=$$

$$=\frac{1}{16}\sum_{j} var\left( p_{ij} \right)+2\sum_{j} \sum_{k>j} r\sqrt{var(p_{ij})}\sqrt{var(p_{ik})}$$

where *n* is the number of repeats in each assay (= 4) in the *k*-th block, *r* is the within-assay correlation between the repeats (assumed to be equal to 0.8), and the double square-root term is assumed to be 0.0005 (average sampling variance of proportions across all assays). The average proportion (over 4 repeats) is simply an arithmetic mean.

To propagate the variance of (aggregated) proportion, we used the delta method, assuming that final fitness was expressed as $W_{j}=ln({\hat{p_{1j}}}/{\hat{p_{0j}}})$:

$$var\left( W_{j} \right)=var\left[ ln\left( \frac{\hat{p_{1j}}}{\hat{p_{0j}}} \right) \right]=\left[ \frac{\partial}{\partial\hat{p_{1j}}}\left( \ln\hat{p_{1j}}-\ln\hat{p_{0j}} \right) \right]^{2}var\left( \hat{p_{1j}} \right)+$$

$$+\left[ \frac{\partial}{\partial\hat{p_{0j}}}\left( \ln\hat{p_{1j}}-\ln\hat{p_{0j}} \right) \right]^{2}var\left( \hat{p_{0j}} \right)=\frac{1}{{\hat{p_{1j}}}^{2}}var\left( \hat{p_{1j}} \right)+\frac{1}{{\hat{p_{0j}}}^{2}}var\left( \hat{p_{0j}} \right)$$

The final effect size was derived as standardized mean difference (Cohen’s *d*) between the evolved and ancestral population’s fitness scores (*n* was always = 4):

$$d= \frac{W_{ee,j}-W_{anc,j}}{s_{pooled,j}}J$$

$$s_{pooled}=\sqrt{\frac{\left( n-1 \right)var\left( W_{ee,j} \right)+\left( n-1 \right)var(W_{anc,j})}{2n-2}}$$

$$J=1-\frac{3}{4\left( 2n-2 \right)-1}\approx0.87$$
