# Supplementary material for: Reproductive system, temperature, and genetic background effects in experimentally evolving populations of Caenorhabditis elegans
Source: PLoS One. 2024 Apr 1;19(4):e0300276. doi: 10.1371/journal.pone.0300276 (PMC10984399; doi:10.1371/journal.pone.0300276)
Supplement: Attachment — Submitted filename: reviews&responses.docx [file pone.0300276.s002.docx]

Dear Dr. Prokop,

The two expert reviewers who evaluated your manuscript agreed that your study was interesting and potentially suitable for publication in PLoS ONE.

They also identified several aspects of data analysis, interpretation, and description of results that require modification.

If you wish to have your manuscript to be further considered by PLoS ONE, please address all of their comments thoroughly. In particular, the claims related to the "genetic background" were seen by the reviewers as insufficiently established.

Please accompany your revised manuscript with a detailed list of how each of the reviewers’ criticisms was addressed by you.


**Response:**

**Dear Dr. Ruvinsky,**

**thank you for handling the manuscript. Below we are our responses to the reviewers’ comments (in bold case). Additionally, we would like to emphasize that we have changed the References section, adding 3 papers which were not cited in the previous version of the manuscript (refs. 38-40 in the revised version) as well as correcting a number of formatting & missing information mistakes which had escaped our attention in the first version of the manuscript.**

**Sincerely,**

**Zofia Prokop (on behalf of all co-authors)**

Reviewers' comments:

Reviewer's Responses to Questions

**Comments to the Author**

1. Is the manuscript technically sound, and do the data support the conclusions?

The manuscript must describe a technically sound piece of scientific research with data that supports the conclusions. Experiments must have been conducted rigorously, with appropriate controls, replication, and sample sizes. The conclusions must be drawn appropriately based on the data presented.

Reviewer #1: Partly

Reviewer #2: Yes

2. Has the statistical analysis been performed appropriately and rigorously?

Reviewer #1: Yes

Reviewer #2: Yes

3. Have the authors made all data underlying the findings in their manuscript fully available?

The [PLOS Data policy](http://www.plosone.org/static/policies.action#sharing) requires authors to make all data underlying the findings described in their manuscript fully available without restriction, with rare exception (please refer to the Data Availability Statement in the manuscript PDF file). The data should be provided as part of the manuscript or its supporting information, or deposited to a public repository. For example, in addition to summary statistics, the data points behind means, medians and variance measures should be available. If there are restrictions on publicly sharing data—e.g. participant privacy or use of data from a third party—those must be specified.

Reviewer #1: Yes

Reviewer #2: Yes

4. Is the manuscript presented in an intelligible fashion and written in standard English?

PLOS ONE does not copyedit accepted manuscripts, so the language in submitted articles must be clear, correct, and unambiguous. Any typographical or grammatical errors should be corrected at revision, so please note any specific errors here.

Reviewer #1: Yes

Reviewer #2: Yes

5. Review Comments to the Author

Please use the space provided to explain your answers to the questions above. You may also include additional comments for the author, including concerns about dual publication, research ethics, or publication ethics. (Please upload your review as an attachment if it exceeds 20,000 characters)

Reviewer #1: This is a very worthwhile study, for a number of reasons, and it is important to publish. Everyone contemplating experimental evolution with C. elegans (or anything else, for that matter) should read and take it to heart. Similarly, anyone interested in the experimental quantification of competitive fitness should read and take to heart. PLoS One seems like an appropriate venue.

As much as anything, the authors demonstrate that the Road to Hell is paved with good intentions. They set out to be the Lenskis of the multicellular world, and they found it's not so easy. Of their hoped-for 200 lines evolved for 200 generations, partitioned into starting isolines, temperature and mating systems, after all is said and done they report results from 35 lines evolved for an average of 127 generations, distributed unevenly across treatments.

**Response: Thank you! This is a pithy summary of our journey from huge ambitions & excitement through a series of humbling reality checks (including mistakes, too). We appreciate your understanding and also feel that the paper is important to publish.**

The first key feature of this work is shown clearly in Figure 4. Looking at the fog-2 ancestor of isoline 8 at 20 deg, the estimates of W cluster tightly within blocks. Similar patterns hold for other ancestors. These lines are (nearly) isogenic, as is the competitor strain**. Thus, the variance in these assays must be due to heritable environmental effects (which are common in C. elegans) in the focal strain, the competitor, or (very likely) both.

**Response: We agree that epigenetic effects are likely important here, and we have now added a short passage on that subject (lines 484-495 in the revised manuscript with tracked changes), although without further investigations (which we have not done) we wouldn’t go as far as saying our data patterns *must* be due to epigenetics. Conceivably, some direct environmental effects operating during the assays & varying among blocks could also have driven these patterns.**

** We are not given any details about the competitor strain beyond "...a green fluorescent protein (GFP)-marked strain was used as a standard competitor". What was the strain? How inbred was it? How was the competitor propagated among blocks? Was a large population cryopreserved in many aliquots at the same time and one aliquot thawed for each assay block? Was the competitor cryopreserved from multiple plates, maybe at different times? These are important details, which we are not told.

**Response: Thank you, this are indeed key details which we should have included, as we now do in lines 216-222 and 318-320 (revised manuscript with track changes). Briefly, GFP allele was introgressed into an isoline derived along and in the same way with the ancestral isolines, banked in many aliquots and thawed, one aliquot at the time, for each block.**

Which leads to the second key feature, which is that isoline is confounded with block(s). Isoline 9, which is the only one of the three that consistently evolved, was assayed in blocks 5 and 6. If for some reason both the ancestor and the competitor strain was compromised in those two blocks but the evolved lines were not – which is certainly possible – the increase in fitness would be a mirage. The fact that isoline 9 in the fog-2/heat treatment is represented by only a single population (E34) does not help matters any.

**Response: The blocks are indeed nested within isolines in our design. The logistic rationale for this was that (i) there was a limit on the number of populations we could assay in a single block due to the workload involved, and (ii) each block had to include ancestral populations, of which there are two (wt & fog) for each isoline. Thus, for each +1 isoline included in a block, we would have +2 ancestral populations in addition to the EE ones. This is why we decided to block the isolines together instead. However, we do agree this has limitations in terms of inference, especially given the small number of blocks per isoline, and small numbers of populations in some treatment groups (fog 24C in line 9 being the worst case). Nevertheless, we do not think these limitations are quite as strong as you suggest. In case of Isoline 9, the scenario you are invoking would have actually required 3 populations (competitor as well as both ancestors: wt and fog) to be compromised in both blocks 5 & 6, relatively to the average of EE populations. While certainly possible, we do not think it is highly likely. Furthermore, the isoline effect was also revealed in the interaction (20C vs. 24C fog populations in isoline 8). We further discuss these issues in response to your related comments below.**

And also, there are only four biological replicates per population, and no more than eight populations per treatment, and sometimes as few as two. Four replicates per population is reasonable if the point is to (say) estimate the among-line variance of a large set of inbred lines or wild isolates. It is noteworthy, and interesting, that the among-block variance is consistently greater than the among-population variance within a block, of which the point estimate is 0.0000 (Table 2). Since the unit of evolution in this experiment was the population, taken at face value, that result seems strange. Either (a) all the populations within a group evolved in identical ways – which could happen if the infinitesimal model of quantitative genetics is true – or (b) there is insufficient power to detect a relatively small component of among-population variance.

**Response: We agree, and we believe scenario (b) is probably much more likely.**

But clearly, whatever the cause of variation among blocks, it is large compared to the variation among populations, and among replicates within populations. Which brings me to the authors headline claim, that there is an "Effect of genetic background", because one isoline (9) appears to respond to selection, whereas the other two do not. Given that these are "isolines", evolution must be due to new mutations rather than the increase in frequency of segregating variants. In essence, the authors are asking us to believe that isoline 9 is more evolvable than the other two. There are three possible (not mutually exclusive) reasons for differences in evolvability: (1) differences in mutation rate, (2) a different distribution of fitness effects (i.e., epistasis), or (3) random chance. The three isolines were constructed by 20 generations of single-worm transfer from an N2 ancestor. So the genetic variation among isolines will be 20 gens of mutation accumulation, plus 9 generations of introgression with fog-2 (or not), plus whatever segregating variation was present in the N2 progenitor. Then, we are asked to believe that populations derived from one of the isolines evolved – all in the same way, see the preceding paragraph – whereas populations derived from the other two isolines did not. Given that N2 is already highly inbred, and that fog-2 is initially in an N2 background, and that 20 gens of MA will introduce about 6-8 new mutations per-genome, I doubt the three isolines are very different genetically. Which is to say, given the large variation among blocks and the confounding of block with isoline, and the sketchy description of how the competitor was handled, I very much doubt that the difference of isoline 9 has anything at all to do with its genetic background. Random chance sounds way more plausible.

**Response: As we now state in the revised manuscript (lines 535-541, tracked-changes version), we agree that random variation is a plausible alternative explanation for the isoline effects we have found in our data and attributed to genetic background; however, with the current data, be believe that jury is still out on these alternatives. First, we agree that genetic differences among the isolines cannot be very large; however, small genetic differences may be impactful biologically (our ongoing sequencing-based study will, hopefully, provide some insight here). Secondly, our model, which controlled for block variance, did show isoline effects as statistically significant. That this significance may be spurious is a fair criticism, given the small number of blocks. In principle, running more blocks would allow a verification – showing whether the observed pattern of differences among isolines disappears (which would mean it was indeed a mirage driven by random chance) or stands up (indicating the role of genetic differences among isolines). Adding a second competitor strain, as you conclude below, would certainly also help. Unfortunately, we were not able to run more assays and will not do so in the future, since (as we explain in our responses to Reviewer 2) we are closing this research program. Thus, in the current version of the manuscript we now simply present the two alternatives.**

So here's the bottom line. This is an important paper for anyone interested in experimental evolution in Caenorhabditis, and I want to see it published. I have experienced most (if not all) of the frustrating aspects of this project in my own 20 years of working with experimental evolution with Caenos, in particular the perfidy of variability among isogenic lines across fitness assay blocks.

**Response: Thank you. As mentioned above, we really do appreciate your understanding of the frustrations associated with this work…**

It is now abundantly clear that short-term heritable epigenetic effects are both ubiquitous and extremely important in C. elegans, and that the problem is squared with competitive fitness assays because the effects apply equally to the competitor strain. In fact, in reading this paper I reached the conclusion that competitive fitness assays should always include two independent competitor strains as a control, which would allow among-block variability to be attributed either to the focal strain or (one of) the competitors.

**Response: We agree, and we have now included the mention of more competitors in Discussion (lines 496-501, manuscript with tracked changes)**

signed,

-Charlie Baer

Reviewer #2: Baran et al. report the results of a massive experimental evolution study in C. elegans. Starting with very little standing genetic variation, the presence of adaptation depended primarily on the starting strains (after 96-165 generations in either selfing or outcrossing lines reared at two different temperatures). This work is a valuable contribution to the literature. I can find no obvious problems with this manuscript (although I do not have the expertise to evaluate the Appendix). Below are my minor comments.

Discussion and motivation. I appreciate the references to the Lenski LTEE. I have also thought that an analogous study in nematodes could potentially be a powerful inroad towards understanding how evolution works in animals. This clarifies my confusion regarding a similar paper that was recently published (Palka et al. 2023 PeerJ)-- the aim was to potentially evolve populations indefinitely (like the LTEE). This leads to my major issue with the discussion. It is largely oriented around the need for replication in biological experiments. I agree with much of this language, and I think replication should be considered in the interpretation.

Yet, I was surprised by how little language was devoted to how the original isolines are different and what might be next for these EE populations. Previous literature has suggested there exists some cryptic genetic variation in "wild-type N2" lines among laboratories (Gems & Riddle 2000 J. Gerontology Series A; Vergara et al. 2009 BMC Genomics).

**Response: These studies have indeed found variation among N2 strains kept at different laboratories; however, our starting N2 population – from which we derived the isolines – was a sample obtained directly from *Caenorhabditis* Genetics Center. Thus, the variation identified in these studies would not be present here.**

What is the extent of genomic variation among the ancestral isolines, and what new mutations emerged in isoline 9 that could potentially drive adaptation in this background?

**Response: Unfortunately, we do not have any answers to these questions at the moment, which is why we have not discussed this issue in the manuscript. Hopefully, however, we will have them in the foreseeable future. We have sequenced the populations (ancestral as well as EE) from all three isolines and we are in the process of analyzing these data. Truth be told, it is going slowly, since we all have now moved on to different projects, so this one is being done as a side thing. However, we will complete it eventually.**

And, what are the future plans for these EE populations?

**Response: Here our answer may be disappointing, but it is, nevertheless, true. Apart from the ongoing sequencing study mentioned above, we do not have any further future plans for these populations. We have moved on to different things. In particular, in case of the PI (Zofia Prokop, writing these words): my research interests have profoundly shifted over the last couple of years, and I am ready to close this research program down.**

Figures. In Figure 4, it might be useful to have a dotted horizontal line to note the schgmean ancestral fitness for each line/temperature/reproduction group. As that is the most important comparison, I was struggling to visualize that key difference across the various panels of this figure.

**Response: Thank you! This was an excellent suggestion, we have now added the lines and we think they indeed substantially improve the Figure.**

Additionally, Figures 4-5 still have default axis ("ln_prop") or legend ("repro.temp") labels that, while recognizable, are disheveled.

**Response:Corrected**

Very minor comments

Line 56. Should this be "Not only do both of these features..." ?

**Response:Corrected**

Line 200. "Apart from the fog-2 sequence..." Also fog-2 should be italicized.

**Response:Corrected**

Line 218-221. Can embryos also pass through these filters?

**Response: They cannot, we have now made it explicit (line 237 in the revised manuscript with tracked changes)**

Line 240-265. How often do fog-2 gene conversion events occur?

**Response: Unfortunately, we do not know. Neither our own data (presented in ref. 35, Antoł et al. 2022, microPublication Biology), not previous publication by other researchers (ref. 34) allow for reliable quantitative estimate of the frequency of these events.**

Also, reference 35 does not have a journal listed.

**Response: Thank you! We’ve corrected it, and while at it, found a number of similar problems in our References list (something had clearly gone wrong there and skipped our attention), so we have now replaced the whole list with a corrected one.**

Line 250. Should this be "met with" instead of "verified by"?

**Response: Changed as suggested**

Line 461. Should this be "wild-type" instead of "wt"?

**Response: Since we are using the “wt” abbreviation throughout (as introduced in Methods, line 204 of the revised manuscript), we would prefer to leave it as is.**

Thanks for sharing this work.

**Response: Thank you for you kind review.**

6. PLOS authors have the option to publish the peer review history of their article ([what does this mean?](https://journals.plos.org/plosone/s/editorial-and-peer-review-process#loc-peer-review-history)). If published, this will include your full peer review and any attached files.

If you choose “no”, your identity will remain anonymous but your review may still be made public.

**Do you want your identity to be public for this peer review?** For information about this choice, including consent withdrawal, please see our [Privacy Policy](https://www.plos.org/privacy-policy).

Reviewer #1: No

Reviewer #2: No
